# Characterization of Solid Fuel Chars recovered from Microwave Hydrothermal Carbonization of Human Biowaste

**DOI:** 10.1016/j.energy.2017.06.010

**Published:** 2017-06-04

**Authors:** Oluwasola O.D. Afolabi, M. Sohail, C.L.P. Thomas

**Affiliations:** a School of Civil and Building Engineering, Loughborough University Loughborough, LE11 3TU, UK; b Department of Chemistry, Loughborough University, Loughborough, LE11 3TU,UK

**Keywords:** Faecal biomass, microwave, hydrothermal carbonization, chars, sanitation, renewable energy

## Abstract

Microwave hydrothermal carbonization (M-HTC) is reported in this study as a viable sanitation technology that can reliably overcome the heterogeneous nature of human faecal biowaste (HBW) and realize its intrinsic energy value. Solid chars produced from the M-HTC process at 180°C and 200°C were characterized to further the understanding of the conversion pathways and their physicochemical, structural and energetic properties. The study revealed solid chars recovered were predominantly via a solid-solid conversion pathway. In terms of yield, more than 50% of solid chars (dry basis) can be recovered using 180°C as a benchmark. Additionally, the carbonized solid chars demonstrated enhanced carbon and energy properties following the M-HTC process: when compared to unprocessed HBW, the carbon content in the solid chars increased by up to 52%, while the carbon densification factor was greater than 1 in all recovered chars. The calorific values of the chars increased by up to 41.5%, yielding heating values that averaged 25MJ.kg^-1^. Thermogravimetric studies further revealed the solid fuel chars exhibited greater reactivity when compared with unprocessed HBW, due to improved porosity. This work strengthens the potential of the M-HTC sanitation technology for mitigating poor sanitation impacts while also recovering energy, which can complement domestic energy demands.

**Highlights**

- Microwave hydrothermal carbonization can convert human biowaste to solid fuel chars
- Physicochemical, structural, energetic and combustion properties of chars are enhanced
- Higher heating value (HHV) of chars recovered increased by up to 41.5%
- HHV of chars — up to 25.6MJ/kg — is greater than that of low-ranking coals/fuels
- Processing human biowaste into solid fuel is promising for energy applications

## 1 Introduction

Two of the key issues facing more than 2 billion people in developing countries are poor sanitation and energy scarcity. Having missed the Millennium Development Goals’ targets by wide margins, achieving access to adequate and equitable sanitation, ending open defecation/untreated faecal wastewater and increasing the share of affordable, renewable clean energy by 2030 now constitute the key targets of the recently adopted Sustainable Development Goals 6 and 7 (1). Annually, an estimated one billion tons of faecal wastewater is generated (2) and as much as 90% of this is discharged untreated (3, 4). Open defecation is still practised by almost one billion people, and about 2.4 billion people still lack access to improved sanitation. (5).The consequences of poor sanitation are pervasive. Open defecation fields (which serve as breeding sites for insects, vectors/disease pathogens), odour nuisance, exposure to human faecal biowaste (HBW) during manual pit emptying and the indiscriminate disposal of this waste into surface water, open drains, near slums and other fragile settlements constitute serious public health and environmental risks. Faecal contamination of drinking water resources is primarily responsible for the high infant mortality rates due to waterborne diseases such as diarrhoea, which kills 700,000 children per year (6). Aside the environmental and health impacts, experts estimate that lack of access to sanitation cost the global economy US$223 billion in 2015 — based on an economic valuation of the costs associated with premature death and loss in productivity, the healthcare costs of the sick and time forgone due to lack of access to improved sanitation (7).

In common with poor sanitation, energy scarcity affects the least well off; an estimated 90% of people in developing economies lack access to reliable energy supplies (8). More than two billion people rely on firewood, charcoal and other related forest biomass to meet domestic energy demands such as cooking and heating (9). Over-collection of firewood and production of wood charcoal are not without environmental impacts — such as, deforestation, ecological deterioration, and high levels of erosion, air pollution and greenhouse gas (GHG) emissions (10). At the same time, recent trends in urbanization and rapid industrialization, especially in developing and emerging economies, suggest availability of energy resources cannot lag behind strategic development plans. Hence, novel strategies and innovative approaches/technologies that can address the twin goals of sanitation and renewable energy generation, especially at the domestic level, are imperative.

HBW — which in this context covers primary sewage sludges, untreated excrement, and fresh or partially digested faecal sludge obtained from cesspits, septic tanks and other related storage media — represents a hazard due to its pathogenic loads, ranging from 10^5^ to 10^11^ faecal coliforms per 100 ml^-1^ (11). Interestingly, however, its chemical composition indicates such waste to be a rich renewable biomass resource of significant value, which can be beneficially exploited, rather than a taboo or problematic waste or expensive liability, as it is conventionally viewed across different cultures around the world. Essentially, HBW is rich in organic matter (more than 90%, dry basis), nutrients (nitrogen [N], phosphorus [P], potassium [K]) and thermal heating value (12). With a generation rate ranging between 120g and 530g.cap^-1^day^-1^ of wet human faeces and 1—1.4L.cap^-1^day^-1^ of urine (12, 13), HBW represents a huge nutrient-rich, sustainable and underexploited resource. Hence, harvesting, recovering and reusing the intrinsic value in HBW using appropriate technologies is sensible — not only in terms of mitigating the impacts of sanitation, but also in contributing to the generation of clean and renewable energy. This aligns with the concepts of ecological sanitation, waste valorization, nutrient recycling/resource looping and the circular economy.

Microwave hydrothermal carbonization (M-HTC), a microwave-assisted thermochemical conversion process, represents a potential sanitation technology that can achieve this; i.e. it is viable to process/treat biowaste and recover valuable end products using this process (14–16). The feasibility of M-HTC technology has been demonstrated to process/treat pure substrates (e.g. cellulose, fructose and glucose) (15, 17, 18) and more complex waste streams such as sewage sludge (14), seafood waste (19), pine sawdust (15), sugarcane bagasse (20), and other lignocellulose waste materials (16, 21, 22). Water, which constitutes up to 95% w/w in HBW, can interact with microwaves, with this electromagnetic interaction causing dielectric heating. The electromagnetic copulation of microwaves with water and other dipolar organics in HBW facilitates rapid and volumetric heating, which can promote novel and faster reaction pathways (12, 15). This is the background theory behind the exploration of a microwave-assisted HTC to process HBW. Conventional HTC process can also be used to process biowaste. When compared, studies have reported that the M-HTC process promotes faster processing time, due to rapid volumetric heating; facilitates higher processing rates, due to relatively lower residence time; improves dewaterability and most importantly consumes less energy (about half the energy required for conventional HTC process) to convert biomass materials to valuable products (16, 23, 24). Hence, using the M-HTC process could potentially improve overall biowaste-processing efficiency. Essentially during any carbonization process, organic biomass is processed under subcritical water conditions at temperatures 160°C to 220°C, under autogenous pressure and in the absence of oxygen. These conditions mimic the natural coalification process, except that a series of simultaneously occurring reaction mechanisms is triggered by the process and these reduce reaction times significantly; they also facilitate the production of a carbonaceous coal-like end product (25, 26). One of the key merits of the process, which makes it attractive in the context sanitation, is its capacity to utilize wet biomass (moisture content > 50%), thereby obviating the need, energy and cost associated with drying biowaste, as obtained during compositing and other thermochemical processes (25). Additionally, using wet HBW essentially makes it ideal for improving sanitation — i.e. for hygienic collection, containment and treatment — as these practices further minimize direct exposure and associated health risks. Preclusion of microbial culture, complete pathogen deactivation at high temperatures, short processing times, a smaller footprint and the absence of fugitive GHG emissions are other factors supporting the M-HTC proposition as a potential sanitation technology (12, 25).

To date, studies involving real faecal waste as a test material using a thermochemical conversion process such as HTC, either for treatment purposes or other related scientific investigations, are very limited. Using faecal surrogates/mixtures is not ideal, as real faecal sludge is different in composition, water absorption properties, chemical and physical properties to such mixtures (12, 13, 27). The adoption of real faecal waste as a test material for the HTC process offers an opportunity to address knowledge gaps in this field. This work is related to that currently being undertaken to develop novel technology based sanitation facility capable of processing HBW to a safe form whilst at same time recover valuable products from it. The focus of this work is on solid fuel char recovery from HBW for potential energy use. Objectively, this work seeks to provide insights into the likely reaction pathways and conversion models associated with the recovery from of solid fuel char from HBW using the M-HTC process. Reporting of the physicochemical, structural, energy and combustion properties of the treated faecal material in this work aims to further address knowledge gaps on char characteristics lacking in the literature and provides useful information for the design, operation and optimization of an HTC-based sanitation facility.

## 2 Materials and methods

### 2.1 HBW feedstock

HBW feedstock used in these study were: primary sewage sludge (SS); faecal sludge simulant (FSS); human faeces (HF) — without urine, flush water or sanitary products; and human faecal sludge (HFS) — including faeces, urine, flush water and tissue paper.

The primary sewage sludge (SS) used for the study was obtained from the primary sedimentation holding tank at Wanlip Sewage Treatment works, Leicester, UK. The SS derives from a catchment area serving a population of 0.5 million people, with mixed domestic and industrial effluent. The moisture content of SS (as received) falls between 95 and 96%.

The human faeces (HF) and human faecal sludge (HFS) used in this study were collected from anonymous donors. For the HF sampling, donors were instructed to separate urine from faeces at the point of defecation. Polythene bags provided to the donors were used to collect faeces. After collection, the faecal specimens were examined to ensure no urine or sanitary products were present. Abnormal faeces were not used in the study. Abnormality was determined through a physical examination and evaluation against the Bristol Stool Chart (28). Only faecal types 3 and 4 were used. Types 1 and 2 are considered to be indicative of constipation, while type 5 is typically low in fibre, and types 6 and 7 are due to diarrhoea. These grouped types have abnormal properties — such as transit time in the human body, diameter and shape configuration, rheology, and chemical and biological compositions, which make them different from *normal* human faeces defecated daily. HFS samples were collected using a portable mobile toilet (‘Porta Potti’) placed in a designated toilet. The HFS samples were also subjected to same physical examination and evaluation against the Bristol Stool Chart, as with the HF samples. Both the HF and HFS samples were homogenized by maceration, and their resultant moisture content adjusted to about 95% (i.e. 5% total solids content) to mimic the faecal sludge characteristics typically found in onsite sanitation facilities (11, 29, 30).

Faecal sludge simulant (FSS) is an artificial faecal sludge prepared from a formulated recipe reported in a study (27) to replicate the chemical composition, water absorption capacity and rheology of real human faeces. After weighing each constituent of the FSS components shown in Table 1, the moisture content of the recipe was adjusted (by mixing uniformly with water) to about 96% (≈4% solids). This was to ensure consistent water-to-solid loading with the SS, HF and HFS samples.

**Table 1:** Faecal sludge simulant (FSS) recipe (dry basis)

Components	Mass (%)	Constituents
Fats	15	Oleic acid (from peanut oil)
Protein	35	Yeast (20%) and miso (15%)
Carbohydrates	30	Bran flakes (5%), psyllium (15%) and cellulose (10%)
Inorganics	5	Potassium chloride (KCl) (2%), calcium chloride (CaCl) (1%), sodium chloride (NaCl) (2%)
Polyethylene glycol	10	Polyethylene glycol
Toilet tissue	5	Toilet tissue

The characteristics of the samples used in this study are summarized in Table 2. All samples were stored between 4^˚^ C and 5^˚^ C, as this temperature range minimizes microbiological decomposition of solids and subsequent loss of volatiles. However, samples were brought to room temperature for all HTC experiments and/or analyses. This is important to ensure a consistent viscosity (which is higher at cold room temperatures) and hence handling and measurement reproducibility.

**Table 2:** Properties of HBW substrates used in this study

Parameters	FSS	SS	HF	HFS
Moisture, MC (%)	95.3	95.2	95.1	94.7
Volatile solids, VS (%)	88.3	73.9	86.7	80.3
Fixed solids, FS (%)	11.7	26.1	13.3	19.7
C (%)	39.1	36.2	47.8	41.4
H (%)	6.2	5.1	6.6	5.8
N (%)	2.6	4.8	5.9	5.3
O (%)*	52.1	53.9	39.7	47.5
pH	4.8-5.0	5.8 - 6.0	7.0-7.5	
Colour	Light brown	Black	Brownish	
Smell	Mild	Foul	Foul	

* Estimated by difference

### 2.2 Microwave carbonization experiments

The amount of unprocessed HBW used for all the carbonization experiments was consistent throughout the study (c.a. 200g). All batch M-HTC experiments were conducted using Anton Paar Multiwave Microwave Labstation (Anton Paar Ltd, Austria) at 2.45GHz frequency, 900W at 10A pulse-controlled power output. Microwave energy supplied to the reactor vessels was controlled by wireless sensors, which monitor internal temperature and pressure inside the vessels and also prevent overheating. In addition, an infrared pyrometer at the base of the microwave cavity measured the temperature in all the reactor vessels and maintained the reactor vessels at ±2°C of set reaction temperature during the M-HTC process. All HBW samples were carbonized at HTC temperatures of 180°C and 200°C and a residence time of 30mins. Guided by literature (12, 18, 26, 29) and preliminary experiments, these HTC process conditions provide enough contact time for carbonisation to occur and ensure pathogen kill. Following carbonization, the reactor vessels were cooled to room temperature and the carbonized materials removed and filtered with a 63μm mesh sieve. The solids, i.e. chars, were dried at 105°C for 24hrs before characterizations. Char yield was estimated using Equation 1.

(1) $$Char\;yield_{\left(dry\;basis\right)}\left(\%\right)=\frac{Wt.\left(dried\right)char}{Wt.\left(dried\right)of\;raw\;feedstock}\times 100$$

### 2.3 Characterizations of unprocessed HBW and solid char products

The unprocessed HBW samples and chars recovered from each substrate at the HTC process conditi ons investigated were characterized using a suite of analytical techniques. Analysis of the moisture content (MC), volatile solids (VS) and fixed solids (FS) was conducted according to Standard Methods 2540G (31), while elemental analysis — i.e. of the carbon (C), hydrogen (H) and nitrogen (N) content — was conducted using a CHN analyser (CE-440 Elemental Analyzer, Exeter Analytical Inc., UK), adopting the ASTM D5373 Standard Test Method. The calorific values, i.e. higher heating values (HHVs), of dried samples were measured using a bomb calorimeter (CAL 2K, Digital Data Systems, South Africa), based on the ISO 1928:2009 Standard. Thermogravimetric (TG) analysis of unprocessed feedstock and char samples was carried out using a thermogravimetric analyser (Q5000IR TGA, TA Instruments, UK). Between 10mg and 30mg of the representative sample was placed in a platinum crucible and heated under atmospheric pressure, with an airflow rate of 50ml.min^-1^. Weight loss and the corresponding weight loss rate, i.e. derivative TG (DTG), of samples were measured continuously under non- isothermal conditions, with a temperature range of 30-900° C at a constant heating rate of 10°C.min^-1^. Surface morphologies of samples were examined on a LEO 1530VP Field Emission Gun Scanning Electron Microscope (FEG-SEM). Imaging was conducted at an accelerating voltage of 5kV primary electron beam current of approximately 200pA. The surface area (m^2^.g^-1^) and pore sizes (nm) of samples were determined using a single point BET nitrogen adsorption analysis on a Micrometrics Tristar Surface Area and Porosity Analyser. Before analysis, 0.2-0.3g of the representative samples were degassed in a vacuum. Analyses of the data and isotherms generated during the analysis were processed to determine the char specific surface area and pore sizes. Surface functionalities were examined using Fourier Transform Infrared (FTIR) spectrometry. The FTIR analysis of all samples was performed using a Shimadzu FTIR-8400S. Samples were run using a Golden Gate diamond ATR (attenuated total reflectance) (Specac Ltd, UK) FTIR spectrometer accessory. Infrared spectra were collected within the 4000-600cm^-1^ regions, with a spectra resolution of 2cm^-1^. To ensure accuracy during this analysis, the background emission spectrum of the infrared (IR) source was recorded and taken into account while collecting the emission spectrum of the IR source from the test material. Background emissions were automatically deducted from each sample emission spectra. Sixty-four (64) scans were collected for each sample.

## 3. Results and discussions

### 3.1 Conversion of HBW during M-HTC based on organoleptic assessment

When assessed against the unprocessed biowastes, the smell, colour and texture of the end products recovered after the M-HTC processing of the four feedstocks were all distinctively transformed. The foul odour associated with unprocessed SS, HFS, HF and FSS was replaced by: a coffee-like smell for SS; an almond-like smell for HFS and HF; and a smell characteristic of burnt oil for FSS. The smell of the end products represents a significant improvement when compared to the foul odour of unprocessed biowastes. The colour of both the carbonized solids (and recovered liquor) after the M-HTC process changed from the brown colour associated with human faeces or sludges to a carbonaceous, coal-like colour. The dried solid chars also appeared denser, friable and could be easily pulverized. The texture of the solid chars further suggested they could be moulded into briquettes or pellets for use as solid fuel.

#### 3.1.1 Mechanisms behind smell and colouration changes

HBW is composed of large macromolecular components: nitrogenous protein compounds, carbohydrates, lipids, minerals and bacterial debris in varying proportions (12, 13, 27). The eradication of the foul odour in carbonized biowastes is primarily due to the thermal hydrolysis of these organic macromolecular components during the M-HTC process. The hydrolytic process facilitates the solubilization of compounds causing foul odour in HBW, such as the nitrogenous benzopyrrole compounds — notably indole and skatole, hydrogen sulphide, the methyl sulphides and other sulphur-containing compounds (32, 33, 34); and renders them nonvolatile, dissolved and trapping them in the liquid phase (35). Other reactions associated with HTC, including aromatization, may also suppress odour (36).

Apart from caramelization reactions, a non-enzymatic browning effect observed on sugars in biowaste processed at elevated temperatures (37), the primary mechanism responsible for the coal-like colouration of M-HTC processed products are the dark-brown coloured complex compounds known as *melanoidins* associated with Maillard reactions (38). M-HTC process conditions/ environment could facilitate the generation of *melcnoidms* through the hydrolysis of large macromolecular components of HBW and subsequent polymerization of mainly low molecular weight intermediates of carbohydrates (reducing sugars, i.e. glucose and fructose) and amino acids via Maillard reactions (38, 39, 40). The Maillard reaction also affects the characteristic smell in the end products, as studies of the Maillard reaction mechanism indicate that the type of smell that will be produced in end products depends largely, among other factors, on the type and nature of proteins present in the feedstock (41). This partly explains the differences in smell observed from the processed biowastes.

The sensory assessment of the smell and colour of the carbonized end products recovered after the M-HTC process showed them to be similar and consistent with thermochemical conversion/transformation processes of organic biomass reported in literature involving other conventional heating methods (35, 42, 43, 44). In essence, these similarities in the organoleptic properties of smell and colour of the end products from the M-HTC of HBW with existing methods, and the characteristic colour and smell change of end products typical of HTC processes, provide preliminary evidence that suggests M-HTC converted HBW to carbonaceous materials.

### 3.2 Conversion of HBW during M-HTC based on SEM micrographs

**Figure 1:**
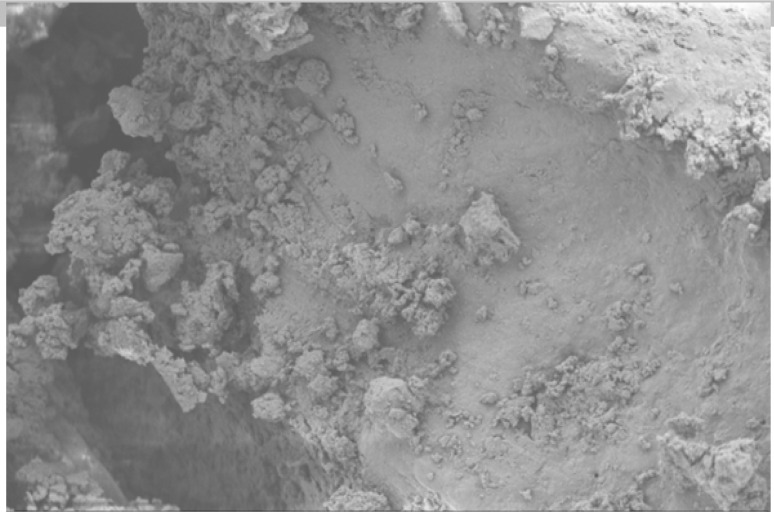
SEM micrograph of unprocessed HF

**Figure 2:**
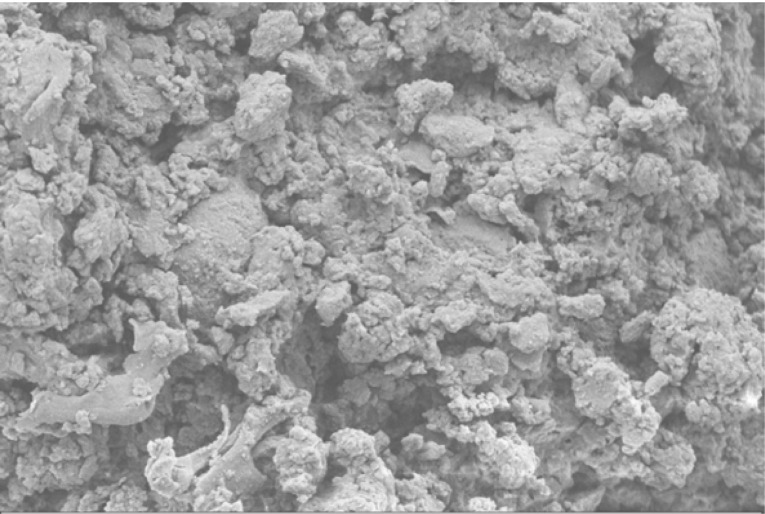
SEM micrograph of HF char

**Figure 3:**
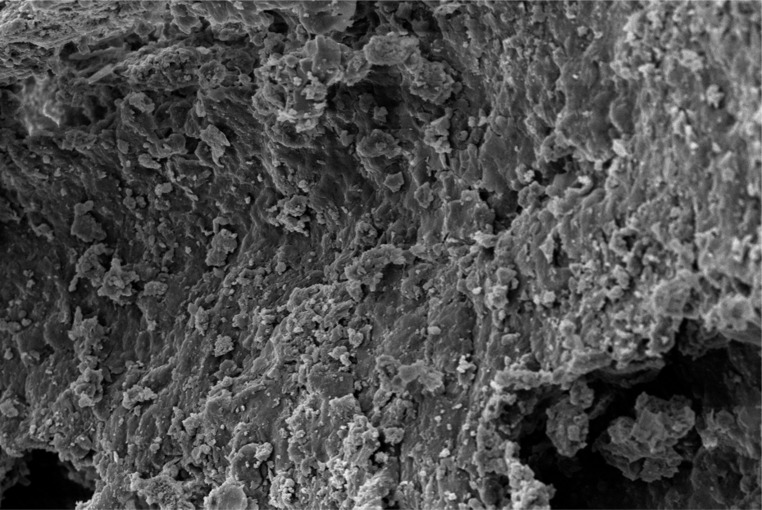
SEM micrograph of unprocessed SS

**Figure 4:**
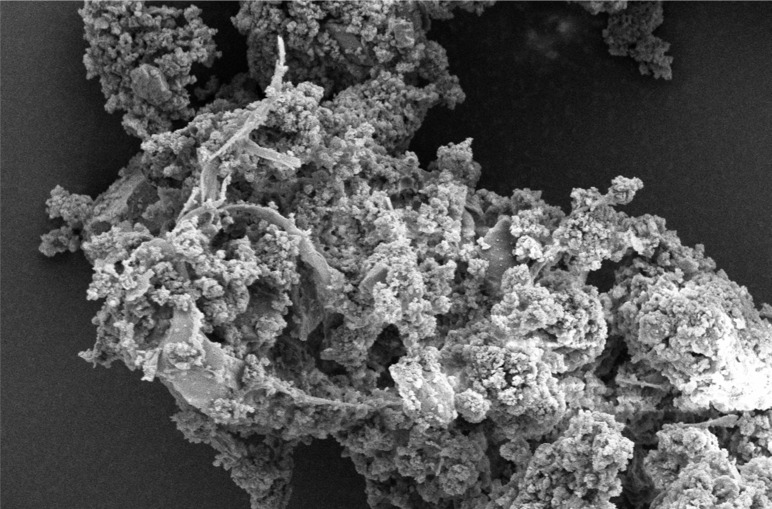
SEM micrograph of SS char

**Figure 5:**
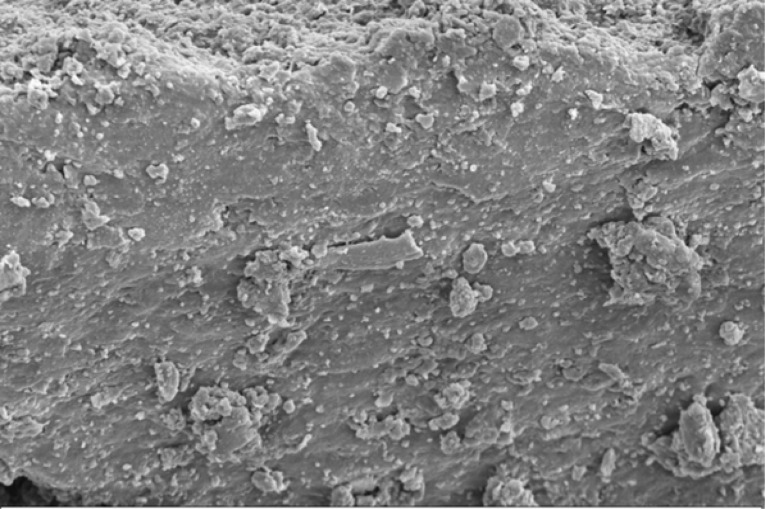
SEM micrograph of unprocessed HFS

**Figure 6:**
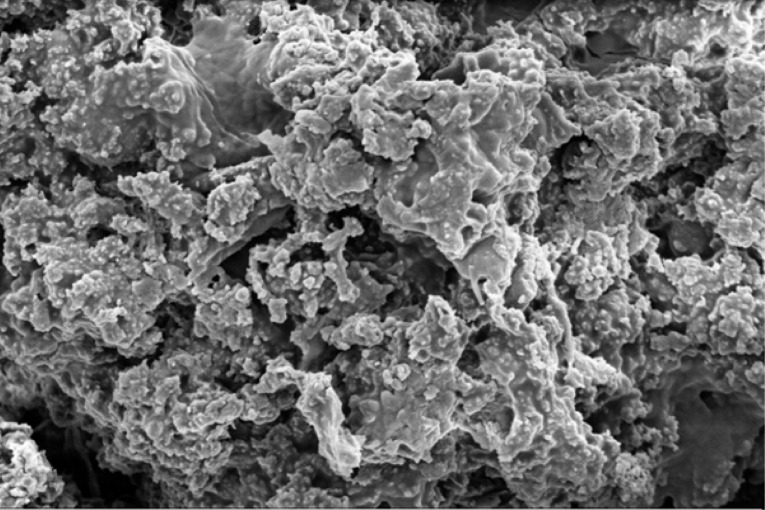
SEM micrograph of HFS char

**Figure 7:**
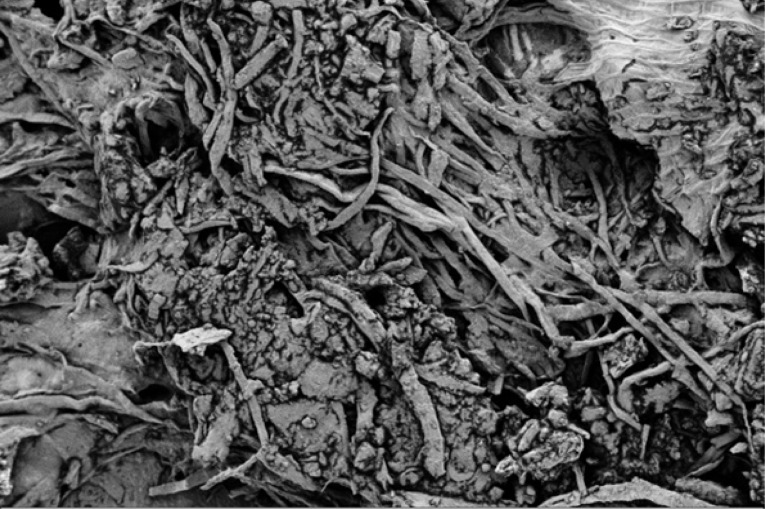
SEM micrograph of unprocessed FSS

**Figure 8:**
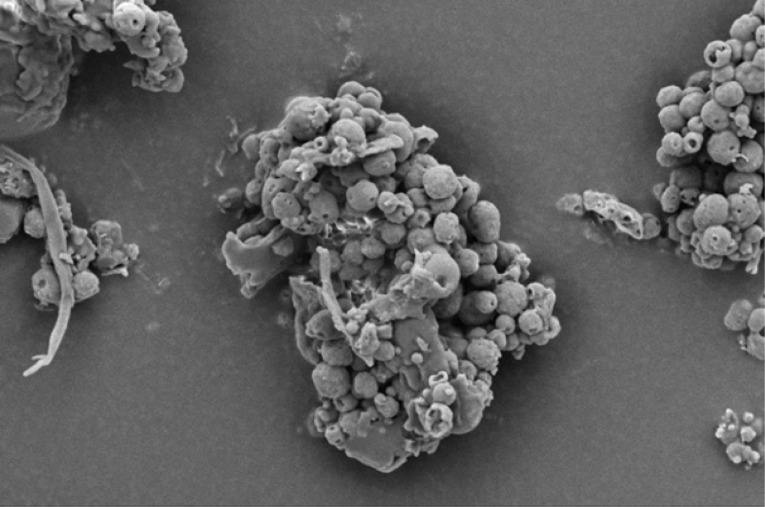
SEM micrograph of FSS char

Scanning electron microscope (SEM) imaging has been used to study potential conversion/formation pathways for char derived from different feedstocks, including glucose and cellulose (15, 18), digested sewage (42) and lignocellulose biomass (45, 46). This technique was also used in the present work to study the changes in morphology of the materials before and after M-HTC at 180°C only. SEM micrographs of the feedstocks (HFS, HF, SS and FSS) and dried chars obtained were studied to investigate likely conversion mechanisms and char formation pathways. Figures 1 to 8 present a series of SEM micrographs (all obtained at x5.00K mag.) of chars recovered from the four biowastes compared with their unprocessed samples. The differences in microstructural morphology are discernible. The information obtained from the SEM micrographs suggests two possible M-HTC char formation pathways from HBW: direct solid-to-solid conversion and induced nucleation pathways.

#### 3.2.1 Direct solid-to-solid conversion pathway

Figures 1 to 8 show the microstructure of HF, SS, HFS and FSS respectively. While unprocessed FSS shows some scattered aggregate of long strands (Figure 7), other unprocessed biowastes exhibit undisturbed flat layers with no discernible porosity (see Figure 1 — unprocessed HF; Figure 3 — unprocessed SS; and Figure 5 — unprocessed HFS).

The morphology of all the chars (Figure 2 — HF char; Figure 4 — SS char; Figure 6 — HFS char; and Figure 8 — FSS char) contains extensive hollow and porous structures, suggestive of devolatilization occurring during the M-HTC process (46, 47). The FSS char (Figure 8) has agglomerations of well-defined spherical features, in contrast to the other chars — which show tampered structures intersected with tunnels. Devolatilization reactions, e.g. decarboxylation and dehydration, have been mentioned with the temperature ranges associated with HTC (25, 48). The hollow-like/porous features which characterize the chars’ microstructures after M-HTC may be partly due to the selective heating of microwaves on water molecules and other polar substances within the unprocessed biowaste materials. This subsequently leads to thermal dissociation of bound water, and decomposition and dissolution of organics within the feedstock structure, leaving the tunnelling effects seen on the char structures. This would imply that the chars were formed by the direct conversion of solid biowaste due to these reactions. This conversion route for unprocessed HBW (except for the FSS sample) agrees with previous studies on char conversion from different feedstocks at about 180—230°C: sewage sludge at 200°C (42); lignocellulosic biomass, e.g. corn digestate and wheat straw, at 230°C (49); and xylose, wood meal and lignin at 225°C (45). It is worth stating that most studies have maintained the solid-to- solid char formation conversion route to be the key or sole pathway of char formation (42, 45, 49).

#### Induced nucleation, polymerization of dissolved intermediates

The SEM micrograph of FSS chars looks entirely different from the others, as it reveals spherical, hollow-like features (see Figure 8), as reported previously (46, 50, 51, 52). The formation of regularly shaped, hollow carbon microspheres from glucose and fructose solutions is well studied and known (53, 54). Such microspheres are formed from induced nucleation and polymerization of dissolved soluble intermediates (mainly furfural compounds, including 5-hydroxymethylfurfural [5-HMF], furfural and 5-methylfurfural) when glucose, for example, is heated to temperatures similar to HTC conditions (52, 55). At 120°C to 140°C, fructose solution undergoes intermolecular dehydration to HMF (50). Another study that also reported the formation of carbon spheres from glucose under HTC conditions of 160°C-180°C, further mentioned that 5-HMF forms after glucose hydrolysis (51). These studies concluded that the HMF intermediates were susceptible to subsequent polymerization/polycondensation reactions, leading to the formation of hollow carbon microspheres. The FSS chars’ formation appears to follow this mechanism, as FSS is a cellulose-based recipe (see Table 1). The thermal hydrolysis of cellulose during M-HTC forms reducing sugar monomers, including glucose, which can exist in isomerism with fructose (42, 56). Intermolecular dehydration of these reducing sugars under HTC conditions leads to the formation of dissolved soluble intermediates, i.e. HMF, which subsequently undergo polymerization — characterized by further intermolecular water loss (dehydration) and forming spherical, hollow char particles similar to those shown in Figure 8.

**Figure 9:**
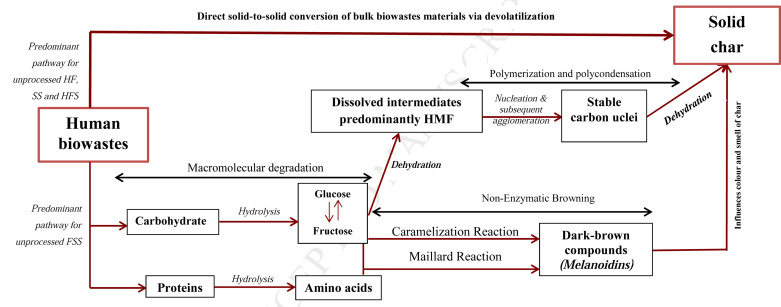
Schematic model of char formation from HBW [adapted from 40, 42]

In essence, both conversion pathways relate to the present study, as modelled in Figure 9. While the SEM micrographs of chars from SS, HFS and HF agree with the solid-to-solid conversion model (as no spherical particles were found), the FSS char micrographs agree with the secondary pathway. What is unknown is the extent to which the conversion pathways contribute to char formation, as the effect of the Maillard reactions (i.e. smell and colour changes due to reactions of reducing sugars monomers from the carbohydrate contents in HBW and cellulose in faecal simulant) are seen across all feedstocks. One study used cellulose as a HTC substrate and argued that both pathways occurred during conversion to char; however, it maintained that at certain temperature ranges <200°C, the solid-to-solid conversion route predominates (57). Although knowledge of char formation mechanisms from complex and heterogeneous substrates, such as human faecal biowaste, is still evolving, the SEM studies conducted here indicate the solid-to-solid conversion route to be the predominant pathway for HBW at the temperature used. Conversion pathways during HTC may also be sensitive to the nature/type of biomass substrate used, as the FSS chars support the second pathway (as evident in the proliferation of hollow microspheres). In summary, SEM studies in the present work further confirm the evidence of thermochemical conversion of HBW during the M-HTC pro cess and further support the hypothesis of char generation under M-HTC.

### 3.3 Structural properties of chars

#### 3.3.1 Porosity and surface area

In order to gain further insight into the structural changes caused by the M-HTC process, the surface area (m^2^.g^-1^) and pore sizes of chars obtained from the M-HTC of each feedstock run at 180°C and 200°C are shown in Table 3.

**Table 3:** Surface area and pore sizes of unprocessed biowastes and their chars

Sample description		BET surface area/m^2^.g^-1^	Pore size /nm	
			Adsorption	Desorption
HFS	Unprocessed	0.9	14.9	11.7
	Char at 180°C	1.7	19.1	16.4
	Char at 200°C	1.3	36.5	30.4
SS	Unprocessed	1.6	29.4	22.7
	Char at 180°C	4.2	25.6	21.81
	Char at 200°C	4.7	23.0	21.4
HF	Unprocessed	0.6	15.5	12.3
	Char at 180°C	1.8	10.0	8.8
	Char at 200°C	0.9	12.2	10.0
FSS	Unprocessed	0.5	14.6	11.6
	Char at 180°C	1.0	9.6	8.2
	Char at 200°C	0.9	12.1	9.9

From Table 3, the pore sizes — ranging from 9.6nm to 36nm — may be classified according to the IUPAC classification as Type 2 pore sizes, *mesopores* 2nm to 50nm (58); these are similar to the pore sizes of HTC chars of sunflower and walnut (59). The char pore sizes were consistent, with their surface areas ranging between 0.9m^2^.g^-1^ to 5m^2^.g^-1^; this was similar to the values reported for chars recovered from microwave pyrolysis of straw pellets and willow chips characterized under BET and mercury porosimetry (60). Literature values for BET surface areas of most HTC chars (derived from conventional heating) for feedstocks such as apricot, sugar bagasse, willow, algal and sewage sludge range between 0.67m^2^.g^-1^ and 14.68m^2^.g^-1^ (61 - 65); these are comparable to the values observed in the present study.

The surface area of the chars was generally greater, by more than 50% in most cases, than that of the feedstock. This can be attributed to *tunnelling effects* caused by heating and the mass transfer processes during the M-HTC process. This corroborates the SEM studies, which revealed enhanced porous features in the chars. This observation also agrees with previous studies, which have reported that the volumetric heating occurring during microwave processing of feedstock not only promotes porosity development, but also facilitates higher porosity in the chars produced — especially when compared with those produced from conventional heating (60, 65). When compared with average surface areas of commercially activated carbon, about 1500m^2^.g^-1^ (66), an activation step will be required if M-HTC chars are intended for sorption studies.

#### 3.3.2 Functional surface analysis of unprocessed biowastes and their chars

FTIR studies were conducted to further investigate the microchemistry of HBW and its chars, and Supplementary Figures S1 show the FTIR spectra of feedstocks and chars recovered at 180°C and 200°C. Tables 4 to 7 summarize the spectral analysis. The interpretations of the FTIR spectra and band assignments were informed by published studies of HTC char produced from sewage sludge, food materials and cellulose (65, 67 – 71) under comparable thermochemical conditions.

Supplementary Figures S1 appear to show that the spectra of chars are a ‘superposition’ of components in unprocessed biowaste. Although some differences in FTIR spectra patterns are discernible from unprocessed biowaste, the main difference is seen in the difference in absorbance band intensities. In most cases, spectra from both unprocessed biowastes and their chars contain several similarities in band peaks, with the intense and broad absorptions in the region 1006cm^-1^ to 1058cm^-1^, assigned to the C-O stretching typical of carbohydrates or polysaccharide-like substances that are expected to be present in HBW (71, 72). Two sharp absorption bands, typically at 2920cm^-1^ and 2850cm^-1^, assigned to C-H stretching due to the aliphatic methylene groups (70, 73) were also present. Other bands at 720cm^-1^, commonly observed in HBW such as sewage sludge, may be associated with long-chain aliphatic compounds with conjugated characteristics bands (68). Another prominent band was observed at 3271cm^-1^ to 3280cm^-1^, due to O-H hydroxyl vibrations. Bands at 1620cm^-1^ to 1629cm^-1^, and conjugated bands at 880cm^-1^ to 700cm^-1^, due to C=C vibrations and aromatic C-H bends respectively, were attributed to the presence of aromatic structures that were indicative of aromatization as a potential reaction pathway during the M-HTC process (65, 72, 74).

When compared, the O-H and N-H stretching at 3330cm^-1^ to 3336cm^-1^, assigned to H-bonded hydroxyl and amino groups, were seen in the chars from SS, HFS and FSS, but not in their feedstocks. A feature at 1535cm^-1^ due to N-H in plane and 1408cm^-1^ N-O band stretching was not seen in the chars, while being present in the feedstocks; this is signified in Tables 4 to 7 as ‘D’ for ‘disappeared’. Such bands are typically bands of protein (secondary amides) present in unprocessed HBW, which are not present in chars due HTC solubilization effects. This phenomenon has been described before in studies on sewage sludge composting (71, 73, 74), further evidence of the solubilization and transfer of nitrogen compounds (primarily from proteins in HBW) from unprocessed feedstocks into the liquor phase during the M-HTC process. There were also drastic changes in the aliphatic and polysaccharide band intensities of unprocessed biowastes and their chars, which provide further evidence of organic decompositions during the HTC process. For example, a decrease in band absorbance intensity at 1000cm^-1^ to 1100cm^-1^ (carbohydrates bands) and 2850cm^-1^ to 2920cm^-1^ (aliphatic) can be seen from Supplementary Figures S1. This may be attributed to the effect of dehydration during the M-HTC process (18). In addition, bands at 880cm^-1^ to 700cm^-1^, corresponding to aromatic C-H bends, were observed in chars but not in unprocessed feedstock, indicating the presence of aromatic structure in chars (65).

These observations are similar to previous studies (71, 75), which noted that the FTIR spectra of organic matter are usually qualitatively similar, but differ in the relative intensity of absorbance bands and specific bands. Such differences can provide clues to likely reaction pathways occurring during a conversion process. From this study, hydrolytic solubilization, dehydration and aromatization reactions can be said to have occurred during M-HTC processing of HBW.

**Table 4:** Assignment of the principal IR absorption bands in the spectra of unprocessed SS and its chars

Location of wave numbers (cm^-1^)			Band assignment of functional group/component
Unprocessed SS	SS chars	Vibrations	
	**3335**	N-H stretch & O-H stretch	Aliphatic secondary amines stretch and hydroxy group
**3275**	**3273**	O-H stretch	Hydroxy group
**2955**		C-H stretch	Aliphatic methylene group
**2920**	**2920**		
**2850**	**2850**			
	**1649**	C=O	Primary amide, carboxylates (H-bonded C=O carbonyl stretch)
**1626**	**1629**	C=O stretch C=C stretch	Carboxylate and conjugated aromatic ring mode
**1620**			
**1535**	**D**	N-H in plane	Secondary amides
	**1452**	C-O stretch	Carbonate ion
**1408**	**D**	N-O stretch	A source of nitrate in unprocessed biowaste
**1381**	**1377**	Methyl C-H bend N-O stretch	Provides indication of long-chain aliphatic compounds Nitrate in solid wastes
**1232**	**1259**	C-O stretch C-N stretch	Carboxylic acids or Tertiary amide
	**1024**	C-O stretch	Characteristic polysaccharide bands for C-O stretching of polysaccharides or polysaccharide-like
**1010**	**1006**		
**796**	**798**	NH_2_ out of plane	Primary amine groups
	**719**	Methylene C-H rocking	Provides indication of long-chain aliphatic compounds
	**657**-**692**	O-H or C-H out of plane bend S-O bend	Skeletal vibrations of hydroxy out of plane bend or C-H bending vibration, as corroborated with bands at 1200-1000, 1600-1300 Inorganic sulphates

**Table 5:** Assignment of principal IR absorption bands in the spectra of unprocessed FSS and its chars

Location of wave numbers (cm^-1^)		Vibrations	Band assignment of functional group/component
Unprocessed FSS	FSS chars		
**3331**	**3336**	O-H stretch	Hydroxy and carbonyl groups characteristic of cellulose-based substrate
**3292**	**3286**		
**3005**	**3005**	C-H stretch	Aliphatic methylene group
**2922**	**2922**		
**2852**	**2852**		
**1743**	**1743**	C=O stretch	Vibrations of carbonyl, esters or carboxyl
**1626**	**1651**	C=C C=O	Presence of aromatic rings, ketones/quinones
**1535**	**D**	N-H in plane	Secondary amides
**1454**	**1452**	C-H bend	Aliphatic methylene group
	**1367**		
**1359**		O-H bend	Aromatic bend
**1315**	**1315**		
	**1278**	O-H bend	Primary or secondary OH in plane bend
**1244**	**1242**	N-H bend Stretches of C-O H-O	Secondary amide
**1203**	**1199**		
**1159**	**1159**		Hydroxyl, ester or ether vibrations
**1059**	**1103**		
**1051**	**1055**	C-O stretch	Polysaccharide bands
**1031**	**1030**		
**893**	**896**	Bends due to N-H C-O	Skeletal vibrations due to secondary amine, carbonate, inorganic sulphates
**804**			
	**696**		
**659**	**663**		

**Table 6:** Assignment of principal IR absorption bands in the spectra of unprocessed HF and its chars

Location of wave numbers (cm^-1^)			Band assignment of functional group/component
Unprocessed HF	HF chars	Vibrations	
**3271**	**3271**	O-H stretch	Hydroxy group
**2920**	**2920**	C-H stretch	Aliphatic methylene group
**2850**	**2850**		
**1626**	**1626**	C=O and C=C stretch	Carboxylate, e.g. quinone and aromatic rings
**1535**	**D**	N-H in plane	Secondary amides
**1442**	**1452**	C-O stretch	Carbonate ion
**1408**	**D**	N-O stretch	A source of nitrate in unprocessed biowaste
	**1379**		A band typically observed for decomposed organics
**1317**	**D**	C-N stretch	Aromatic primary and secondary amides
**1236**	**D**		
**1033**	**1058**	C-O stretch	Typical carbohydrate or polysaccharide bands
	**1014**		
	**881**	C-H bend N-H bend	C-H bending vibrations indicating the presence of adjacent aromatic hydrogen in biochar samples and potentially N-H wag
	**779**		
	**721**		
**696**	**700**		
**667**	**665-9**	S-O bends	Inorganic sulphates

**Table 7:** Assignment of principal IR absorption bands in the spectra of unprocessed HFS and its chars

Location of wave numbers (cm^-1^)		Vibrations	Band assignment of functional group/component
Unprocessed HFS	HFS chars		
	**3335-6**	N-H stretch & O-H stretch	Aliphatic secondary amines stretch and hydroxy group
**3273**	**3276-81**	O-H stretch	Hydroxy group
**2918**	**2918**	C-H stretch	Aliphatic methylene group
**2850**	**2850**		
	**1741**	C=O stretch	Esters and carboxylic acids
	**1658**	N-H bend C=O stretch	Amide
**1626**			Carboxylate
	**1579-85**	N-H bend N-O	Secondary amide or nitro-compounds
**1548**			
**1444**	**1452**	C-O stretch	Carbonate ion
**1408**	**D**	N-O stretch	A source of nitrate in unprocessed biowaste
	**1367-9**	N-O stretch	Nitrate source
**1315**	**1315**	C-O C-N stretch	Carboxylic acids Secondary amines
	**1274**		
**1244**	**D**	N-H bend C-O stretch	Secondary amides Alcohols
	**1201-3**		
	**1099**		
	**1053**	C-O stretch	Very consistent carbohydrate or polysaccharide-like bands
**1049**			
**1030**	**1028**		
**896**	**896**	C-H bend	C-H bending vibrations, indicating the presence of adjacent aromatic hydrogen in biochar samples and potentially N-H wag
	**717**		
	**702**	N-H bend	
**657**	**661-3**	S-O bends	Inorganic sulphates

### 3.4 Yield and elemental properties of HBW chars

Table 8 presents char yield and carbon properties of chars recovered at each carbonization temperature from all the HBW samples carbonized in this study.

**Table 8:** Yield, elemental analysis and carbon properties of chars

Parameters	FSS		SS		HF		HFS	
	180°C	200°C	180°C	200°C	180°C	200°C	180°C	200°C
Yield	53.5	47.2	54.8	51.4	51.5	47.6	53.3	48.4
VS (%)	94.7	93.1	58.5	55.3	79.2	75.5	78.9	76.4
FS (%)^a^	5.3	6.9	41.5	44.7	20.8	24.5	21.1	23.6
C (%)	55.6	59.6	38.8	39.5	53.9	56.1	47.4	49.6
H (%)	6.9	7.2	5.1	5.0	6.9	7.1	6.1	6.4
N (%)	2.0	1.3	2.7	2.4	2.6	2.0	1.8	0.8
O (%)^b^	35.5	31.9	53.4	53.1	36.6	34.8	44.7	43.2
Cdf^c^	1.42	1.52	1.07	1.09	1.13	1.17	1.14	1.20
Ceff ^d^ (%)	42.2	52.4		< 10	12.8	17.4	14.5	19.8

^a^ FS provide an indication for the ash content based on Standard Method used in this work

^b^ by difference

^c^ Carbon densification factor, $C_{DF}=\frac{C\left(char\right)}{Carbon\left(dried\;unprocessed\;biowastes\;solids\right)}$

Carbon content increment, $C_{EEF}=\frac{C\left(char\right)-C\left(unprocessed\;HBW\right)}{C\left(unprocessed\;HBW\right)}$

Char yield from the HTC process is sensitive to the type, nature and compositional characteristics of the feedstock, including initial solid loading/moisture content and carbonization temperature, among other factors (25). This may explain the yield observed from all substrates, although slight differences in solid loading must be noted (see Table 2). The range of char (yield) recovered in the present study was similar to that reported in other studies (15, 16, 20), which all involved microwave as the heating source, although with different feedstocks and pre-treatments. More than 50% conversion efficiency of biowaste to solid chars from the M-HTC process is feasible using 180°C as a benchmark. Remarkably, M-HTC initiated changes in the elemental composition of chars recovered from unprocessed biowaste (compare Tables 2 and 8). Changes in elemental composition were observed to be dependent on carbonization temperature. An increase in the carbon content of chars, and a corresponding decrease in the oxygen content, was observed for chars recovered at the carbonization temperatures investigated. Percentage carbon increment due to carbonization effects (see C_EFF_ in Table 8) appeared to be strongly dependent on the nature of the feedstock. SS yielded less than a 10% increase, while the values for HFS, HF and FSS increased significantly, especially at 200°C. Hence 200°C would appear to be the most effective carbonization temperature for HBW in terms of carbon content increment. The carbon densification factors of the chars, which were greater than 1 in all cases, were comparable to the values in literature, which range between 1 and 1.8 (76, 77). In essence, M-HTC increased the carbon content and carbon densities of the chars.

#### 3.4.1 Molar ratio: H/C and O/C

**Figure 10:**
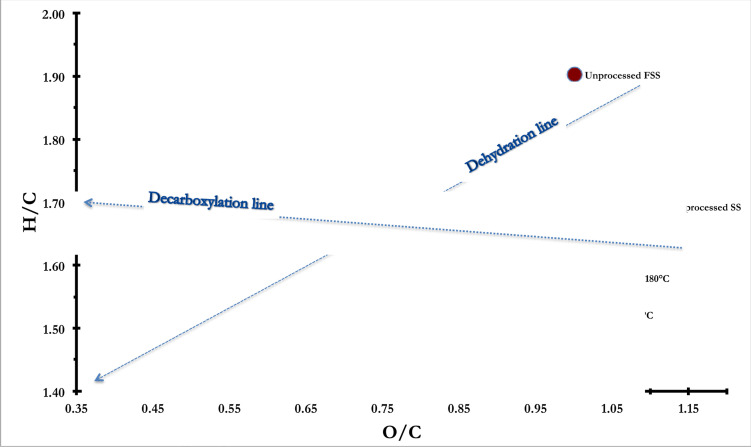
Molar ratio H/C against O/C of unprocessed biowastes and their chars at different carbonization temperatures

Molar ratios of H/C and O/C were estimated from elemental compositions, and were analysed with a Van Krevelen diagram to further understand the reaction pathways involved during the M-HTC of each biowaste. Van Krevelen diagrams allow the delineation of reaction pathways: a line at a slight angle to the x-axis usually represents a decarboxylation pathway, while diagonally drawn lines usually denote a dehydration pathway, as shown in Figure 10.

From Figure 10, the M-HTC of unprocessed biowastes suggests that they are predominantly governed by dehydration reactions. This also supports the discussions on biowaste conversion mechanisms and FTIR studies. Note how the H/C ratio decreases with increasing HTC temperature; this is especially pronounced for chars recovered from HFS and FSS. Decreasing O/C trends can also be seen in Figure indicative of decarboxylation occurring during carbonization, especially for chars recovered from FSS – where O/C ratios decreased from 1.00 in unprocessed feedstock to 0.39 at 200°C. HF and HFS also show slight changes in O/C ratios, reducing from 0.62 to 0.47 and from 0.85 to 0.65 respectively. These observations follow on from previous studies involving the carbonization of glucose, cellulose, starch, sucrose and sewage sludge under conventional processes (57, 77, 78, 79).

### 3.5 Higher heating value (HHV) and combustion behaviour of chars

#### Higher heating values and energetic properties of HBW chars

The calorific value, also known as the higher heating value (HHV), is an important characteristic of chars that enables the determination of key energy parameters – such as improved calorific value, energy densification and energy yield – for comparative assessment with unprocessed biowaste and conventional fuels. Table 9 provides a comparison of the calorific values of chars recovered from each biowaste, and indicates how the calorific values of chars are sensitive to the feedstock and carbonization temperature investigated.

**Table 9:** Calorific value and energy properties of unprocessed biowastes and chars from all feedstocks at different carbonization temperatures

	FSS			SS			HF			HFS		
	Unprocessed	180°C	200°C	Unprocessed	180°C	200°C	Unprocessed	180°C	200°C	Unprocessed	180°C	200°C
HHV/MJ.kg^-1a^	17.1	23.8	24.2	15.3	16.0	16.4	19.5	24.9	25.6	19.3	24.1	25.2
EEF^b^	-	1.39	1.42	-	1.05	1.07	-	1.28	1.31	-	1.25	1.31
Ey / (%)^c^	-	74.4	67.0	-	57.5	55.0	-	65.9	62.4	-	66.6	63.4
CALvi / (%)^d^	-	39.2	41.5	-		< 7	-	27.9	31.5	-	24.9	30.6

^a^ Dried samples

^b^ Energy enrichment factor, $EEF=\frac{HHV\;of\;dried\;chars\;solids}{HHV\;of\;dried\;raw\;biowastes\;solids}$

^c^ Energy yield, $E_{Y}=EEF\;x\;char\;yield\left(\%\right)$

^d^ Calorific value improvement $\left(CAL_{\mathrm{VI}}\right)=\frac{\left(HHV\;of\;dried\;chars-HHV\;of\;dried\;raw\;biowaste\right)\;}{HHV\;of\;\;raw\;biowaste}\times 100$

While FSS and SS behave differently, the values obtained for chars from HF and HFS at 180°C and 200°C are similar. Calorific values were observed to increase slightly over the two carbonization temperatures investigated, and for all the chars recovered the highest HHVs were obtained at 200°C. The effect of M-HTC on HBW feedstock was observed to generate significant calorific value improvement, up to 41.5% for FSS, 31.5% for HF and 30.6% for HFS. HF and HFS chars yielded the highest calorific values, averaged at 25MJ.kg^-1^, which is greater than those for low-rank fuels such as peat (13.8-20.5MJ.kg^-1^), lignite (16.3MJ.kg^-1^), some grades of bituminous coal (17-23.25MJ.kg^-1^), charcoal (23MJ.kg^-1^) (10, 80, 81, 82) (see Table 10). This suggests the use of these chars either as a stand-alone solid fuel for domestic energy consumption (e.g. for cooking and heating) or as one that can be cocombusted with other fuels of similar heating value.

**Table 10:** Comparing heating values (65, 82, 87, 88)

Fuels	Grades of coal	Corn stalk / Stover	Sugarcane bagasse	Softwood wood	Municipal solid waste	Refuse derived fuel	FSS	HF	HFS	SS
HHV* / MJ.kg^-1^	17 to 28	17.6 to 18.5	17.3 to 19.4	18.6 to 21.1	13.1 to 19.9	15.5 to 19.9	17.1 to 24.2	19.5 to 25.6	19.3 to 25.2	15.3 to 16.4

Similar observations of increased heating value of biowastes after pyrolytic processing have been reported in many studies, with many substrates and heating sources (20, 83, 84). For example, reported calorific values of chars recovered from wastewater sludge range from 14.4 to 27.2MJ.kg^-1^ (65, 79, 85, 85) and are comparable to the HHVs obtained for all chars recovered in the present study. Energy densification — as indicated by the energy enrichment factor (EEF — see Table 9) — of all chars recovered from all unprocessed biowastes ranged from 1.05 to 1.41. Similar energy densification ratios were reported for HTC chars produced from municipal solid waste (MSW) (1.01 to 1.41 [86]) and wood- based substrates (1.11 to 1.43 [83]). This is evidence that M-HTC appears to promote energy densification in chars. EEF was also observed to increase slightly with increasing temperature, with FSS recording the highest densification (1.41 at 200°C). Energy yield, which provides a means for assessing the energy recoverable from chars, ranged between 55% and 74% and decreased over the carbonization temperature investigated for all feedstocks — primarily due to the reducing char yield.

### 3.6 Combustion behaviour of unprocessed HBW and their chars

Thermogravimetric (TG) and derivative TG (DTG) analyses were conducted to further understand the combustion behaviour and how unprocessed biowastes and their chars compare when combusted for energy recovery. They also provide an assessment of the impact of the M- HTC conversion process on combustion profiles and the thermal characteristics of solid fuel chars. Supplementary Figures S2 show the TG-DTG profiles of unprocessed biowastes and their chars carbonized at 180°C and 200°C, while Table 11 summarizes key results of the thermogravimetric analyses (TGAs).

**Table 11:** Combustion parameters of unprocessed biowastes and chars

Sample descriptions	IT /°C	^a^ PT /°C	^b^ PT /°C	BT/°C	% BT
Unprocessed	138.8	272.8	463.7	618.7	72.9
SS Char at 180°C	127.0	219.5	278.2	525.6	67.2
Char at 200°C	128.6	205.5	249.7	507.3	60.5
Unprocessed	131.8	281.8	446.7	543.9	88.9
FSS Char at 180°C	159.6	268.3	404.4	502.7	95.8
Char at 200°C	156.0	263.6	372.4	477.8	95.9
Unprocessed	151.1	269.8	489.1	567.2	87.2
HF Char at 180°C	137.9	267.9	392.6	527.2	80.4
Char at 200°C	148.3	255.6	375.2	524.4	83.8
Unprocessed	140.9	271.2	442.8	577.2	86.7
HFS Char at 180°C	146.0	281.9	383.0	522.4	82.6
Char at 200°C	117.9	281.9	380.4	510.5	78.5

Where IT — initial temperature where devolatilization starts

PT ^a & b^ — peak temperature on the DTG profile, corresponding to devolatilization and burning phases respectively

BT — burnout temperature

% BT — percentage weight of material combusted after BT

From Supplementary Figures S2, the relatively slow heating (10°C.min^-1^) of samples in air by TGA suggests three phases of combustion behaviour of unprocessed biowastes and chars: drying, decomposition/devolatilization and burning/ashing. The temperature of the first stage ranged from ambient room temperature at the start of the analysis to about 150°C, corresponding to moisture loss via evaporation and/or dehydration. Some volatiles might also have been evaporated, contributing to the weight loss observed at this phase (89). After the drying phase, thermal decomposition resulted in devolatilization (i.e. weight loss via volatile release) of materials as the temperature profile increased. This stage is generally associated with the decomposition of the organic content of material (90) — (see peaks displayed from the DTG curve in Supplementary Figures S2 of both the unprocessed biowastes and chars). For unprocessed biowaste, this phase started after drying at about 150°C and ended around 340°C for SS, between 350°C and 360°C for both HF and HFS, while for FSS, the phase ended around 413°C. For chars, the temperature ranges associated with this phase were lower when compared with their unprocessed biowastes. This suggests chars were more easily thermally degraded, as the phase ended at less than 300°C for most chars. Differences in peak temperatures, which corresponded to maximum weight loss at this phase, were analysed — as indicated as PT^a^ in Table The final stage, i.e. burning in air (O_2_ atmosphere) and ashing, began after completion of the second stage. Once again this phase ended at lower temperatures (see BT column in Table 11) when the chars were compared with their unprocessed biowastes.

Based on the TG-DTG analyses, M-HTC results in marked differences in the combustion behaviour of chars recovered from unprocessed biowaste. These differences were as follows:

- A distinctive DTG combustion profile and differences in the amount of starting material combusted.
- An increase in carbonization temperature influenced the combustion behaviour of chars, as it tended to make chars more reactive during the decomposition phases.
- Chars exhibited a greater reactivity-to-combustion profile along the TG temperatures than unprocessed biowastes, as their peaks shifted toward lower values of TG temperature and they showed lower burnout (BT) temperatures. These agreed with the textural characteristics and increased porosity of chars reported earlier. Improved porosity enhances air distribution during combustion, which furthers explains the greater reactivity of chars — as observed from the TGA studies.
- Maximum weight loss was recorded during the second phase for chars, while for unprocessed biowaste this occurs mostly in the third phase. This suggests lower temperature regimes can be used to harvest energy from chars as compared with their materials.

## Conclusion

Microwave hydrothermal carbonization (M-HTC) was investigated in this study as an efficient alternative technology capable of addressing the challenges of poor sanitation, which still claims the lives of many in middle- and low-income territories. The study demonstrated that the M- HTC process could transform HBW into a safe form and also realize intrinsic value from that waste. Comparative sensory assessment and SEM studies of HBW before and after the M-HTC treatment provided evidence that indicates M-HTC reliably overcomes the heterogeneous nature of HBW, converting it into new end products that are distinctive in odour (M-HTC completely eradicated the foul odour associated with unprocessed HBW), appearance (colour change and texture) and microstructures. The complete transformation of the foul odour associated with HBW could improve public acceptance, and hence promote M-HTC’s potential use as a mobile processor, representing a safer alternative to the current faecal emptying approach in developing economies. Thermal hydrolysis of macromolecular components in HBW and subsequent chain reactions during the M-HTC process were responsible for the colouration and smells produced from the carbonized biowaste. The conversion model of unprocessed HBW into carbonaceous solid chars was observed to include a combination of solid-to-solid conversion and induced nucleation pathways. The solid-to-solid conversion pathway was observed to be the predominant pathway for all substrates studied, except in the case of the synthetic faecal simulant.

M-HTC of HBW may be used to generate energy in a sustainable manner, for chars are a potential clean energy resource. The handling characteristics of the chars obtained from HBW in the present study are suitable for compaction into briquettes, which can be combusted as solid fuel for domestic consumption. The CHN studies indicated the reduction of N-content in solid chars. This was further buttressed by FTIR studies, which showed the disappearance of specific peaks at 1408cm^-1^ (due to N-O band stretching) and 1535cm^-1^ (due to N-H in plane), associated with protein components found in unprocessed waste but not in the chars. Further, discussion of the mechanism underpinning odour eradication indicates that that the removal of sulphur (S)- containing compounds by M-HTC may be predicted. The removal of N, and potentially S, enhances the suitability of chars for combustion. SEM and BET studies indicate that M-HTC enhances char porosity by more than 50%, improving the combustion reactivity of char — because higher porosity causes more active air distribution. This observation is supported by the observed elevated reactivity of chars during the TGA analysis, such as the decrease in combustion burnout temperatures. HHV results for the M-HTC generated chars from HBW are comparable to those of conventional fuels, which opens up the potential for co-combustion.

Further work required in this area includes, for example, co-blending chars from HBW with conventional fuels, such as coal, and assessing their solid fuel reactivity behaviour and combustibility index. Economic evaluation and sustainability studies of a HTC-based sanitation technology for valuable resource recovery also need to be carefully assessed. Such studies should be informed by more operational, performance development studies of the microwave technology outside laboratory applications; technical and scalability lessons from prototype development, pilot scale experimentations as well as relevant market intelligence studies especially in the context of deploying such technology based sanitation facility in Low and Middle Income countries.

In essence, the M-HTC processing of HBW does more than manage a faecal sludge/sanitation problem, i.e. it has the potential to yield products that are especially useful in energy applications. This can propagate changing views on HBW as a sustainable resource and improve interest for integrating the technology with existing sanitation systems, or encourage design and development of sanitary facilities - either as stand-alone or mobile processing units - that will promote energy recovery from HBW.

## Figures and Tables

**Figure A:**
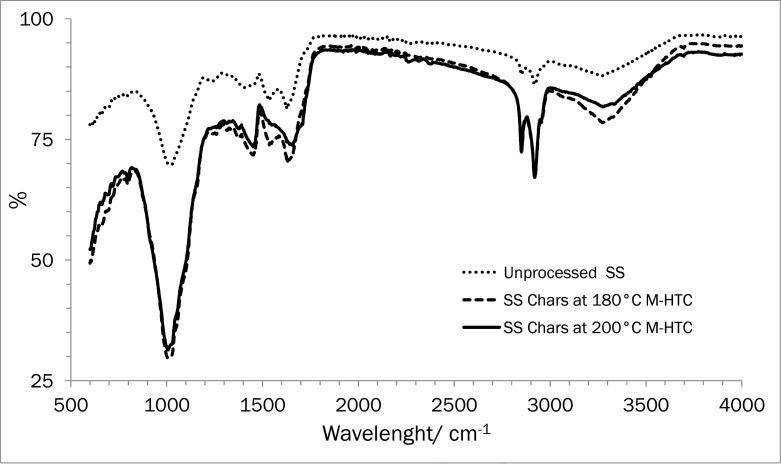
FTIR spectra of unprocessed SS and carbonized chars at 180°C and 200°C

**Figure B:**
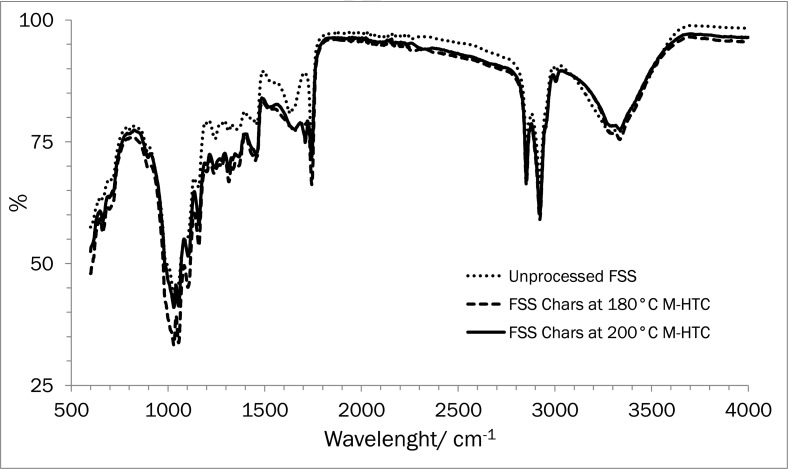
FTIR spectra of unprocessed FSS and carbonized chars at 180°C and 200°C

**Figure C:**
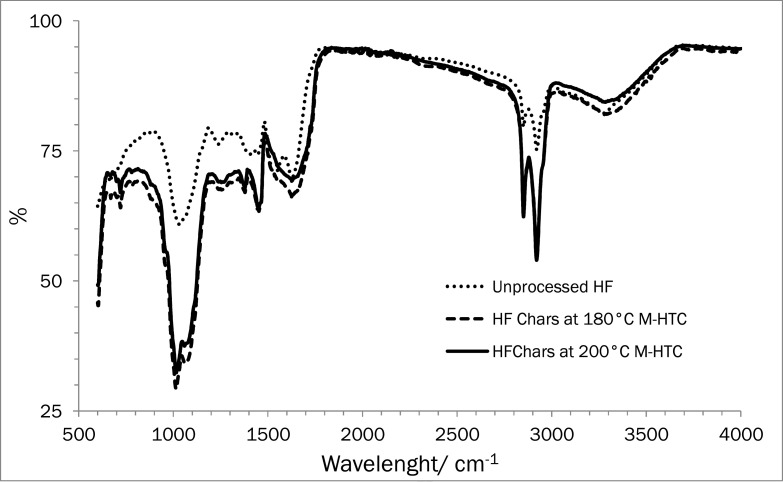
FTIR spectra of unprocessed HF and carbonized chars at 180°C and 200°C

**Figure D:**
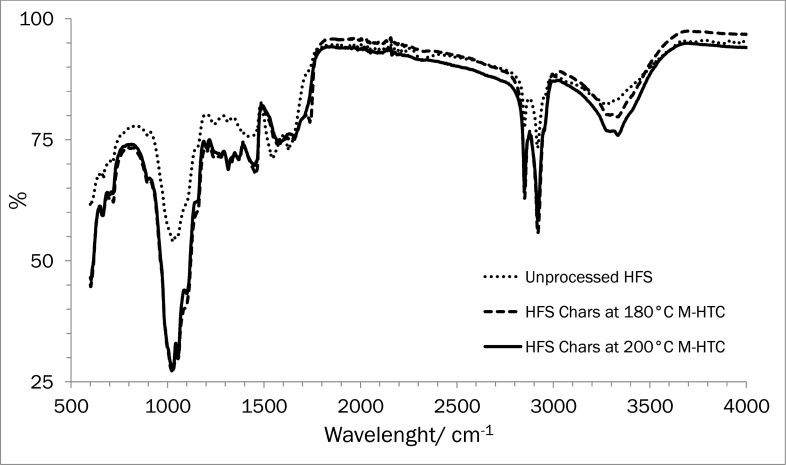
FTIR spectra of unprocessed HFS and its carbonized chars at 180°C and 200°C

**Figure A:**
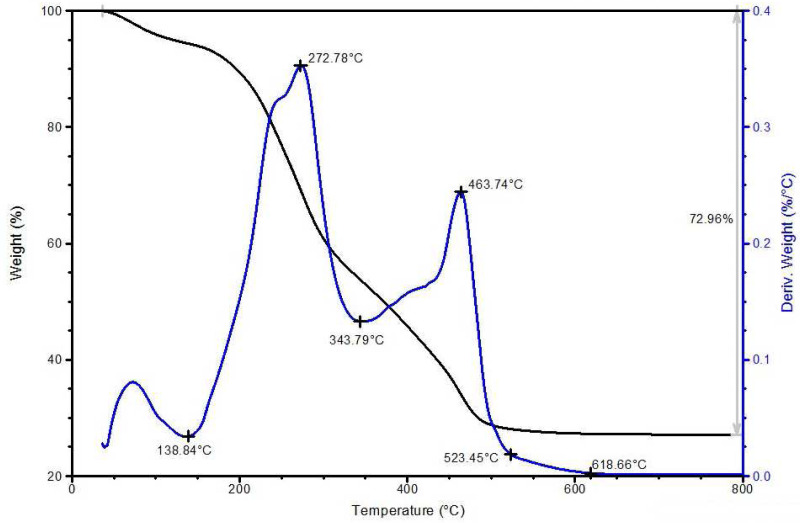
TG/DTG profile of unprocessed SS

**Figure B:**
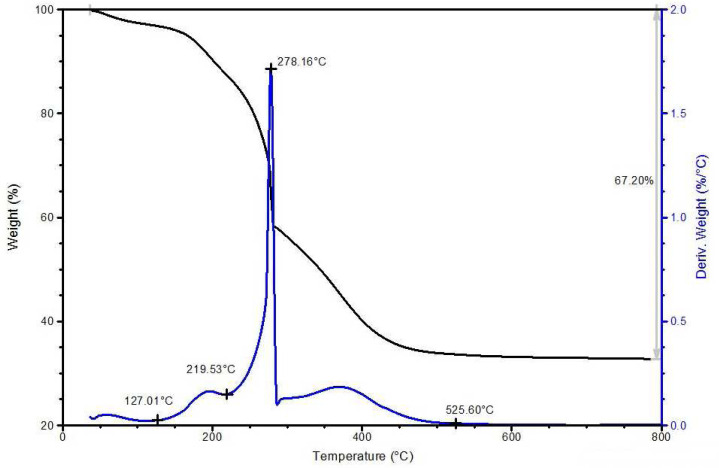
TG/DTG profile of SS char at 180°C

**Figure C:**
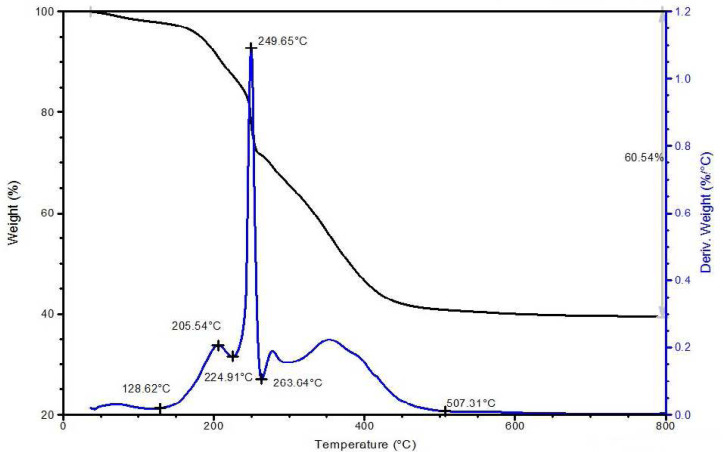
TG/DTG profile of SS char at 200°C

**Figure D:**
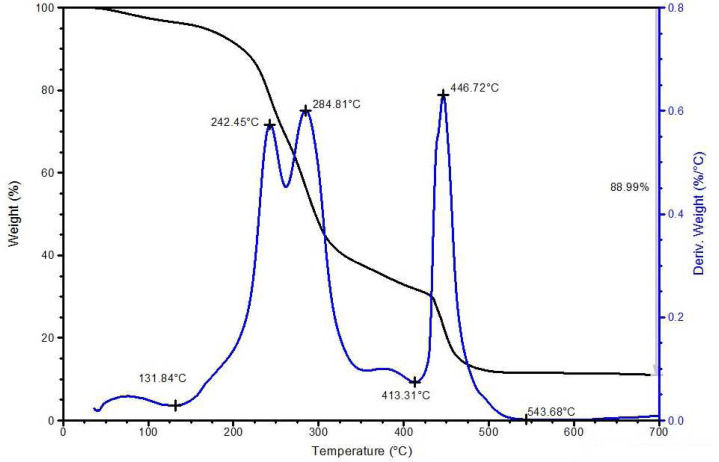
TG/DTG profile of unprocessed FSS

**Figure E:**
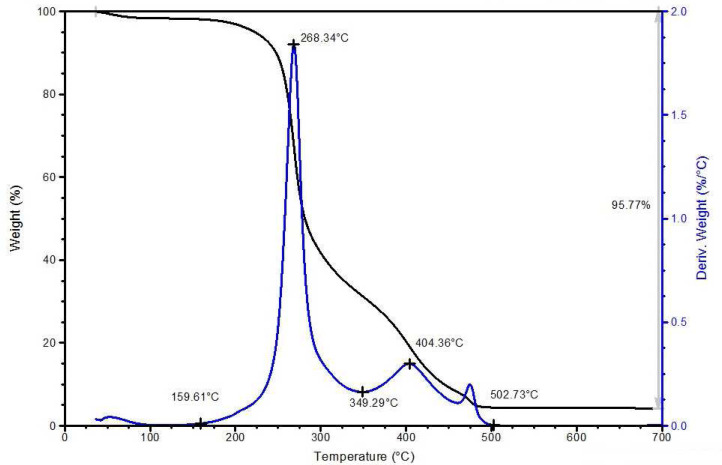
TG/DTG profile of FSS char at 180°C

**Figure F:**
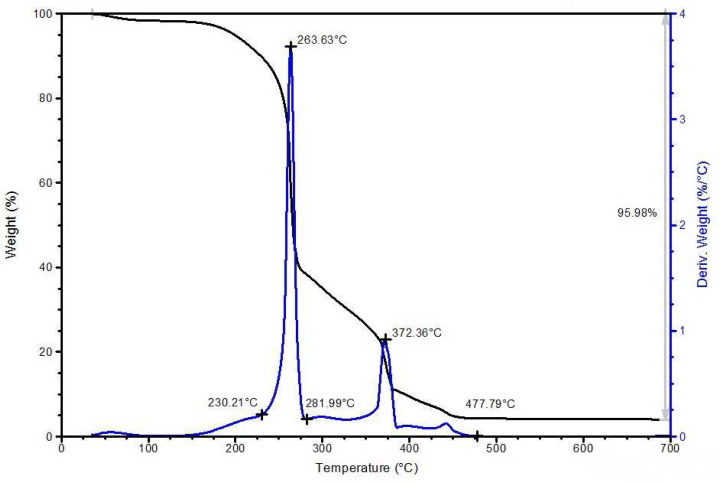
TG/DTG profile of FSS char at 200°C

**Figure G:**
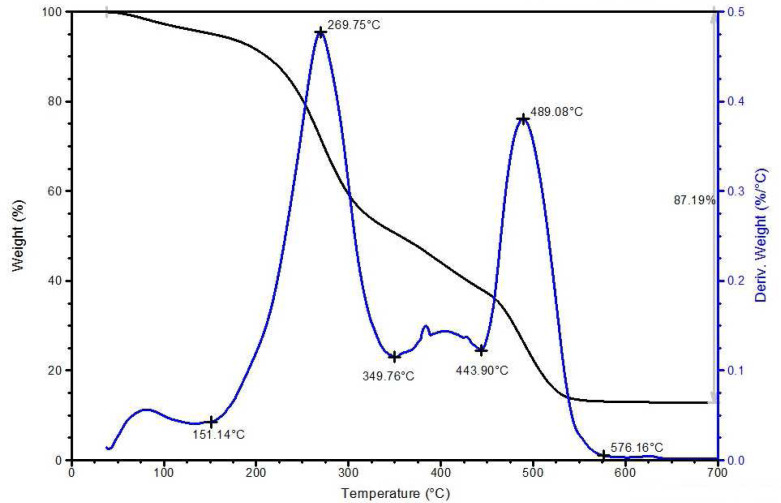
TG/DTG profile of unprocessed HF

**Figure H:**
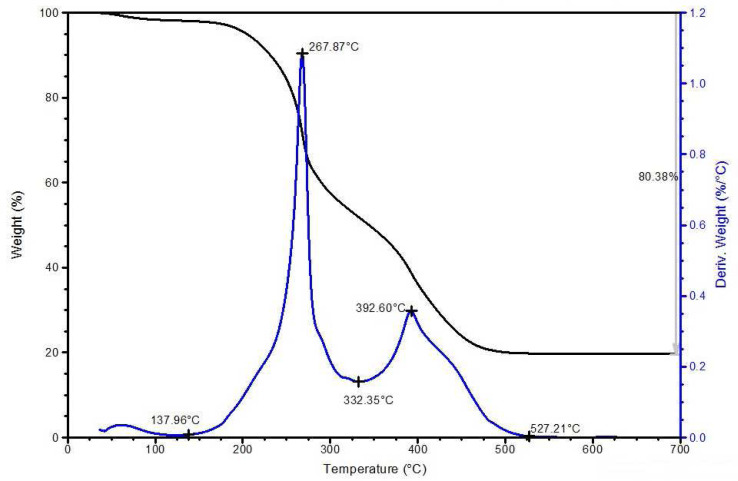
TG/DTG profile of HF char at 180°C

**Figure I:**
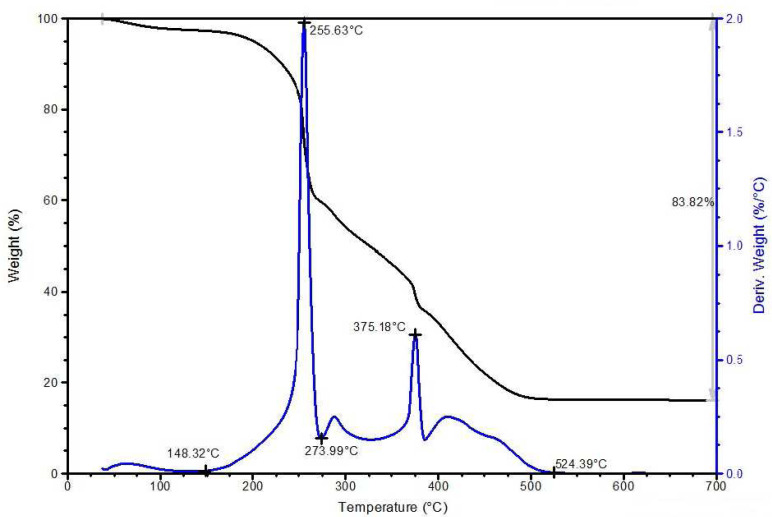
TG/DTG profile of HF char at 200°C

**Figure J:**
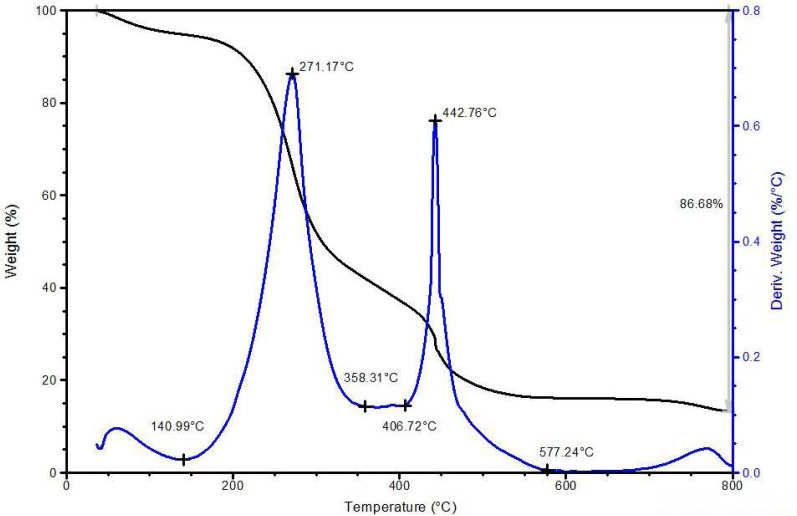
TG/DTG profile of unprocessed HFS

**Figure K:**
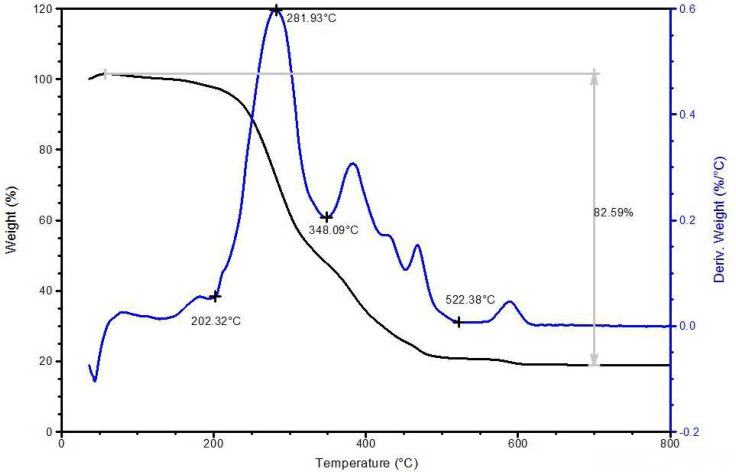
TG/DTG profile of HFS char at 180°C

**Figure L:**
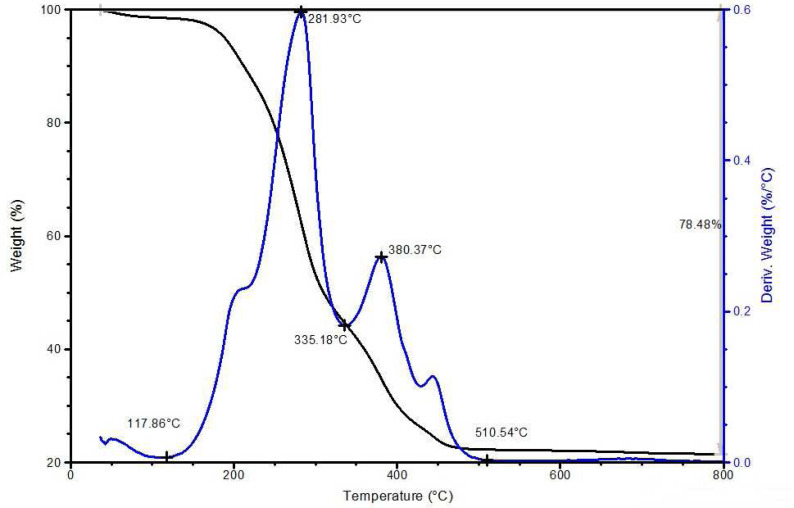
TG/DTG profile of HFS char at 200°C
